# Validation of the Emotional Literacy Program in Croatian Elementary Schools

**DOI:** 10.3390/ijerph18126279

**Published:** 2021-06-10

**Authors:** Tamara Mohorić, Vladimir Takšić, Ana Ćosić Pilepić

**Affiliations:** Department of Psychology, Faculty of Humanities and Social Sciences, University of Rijeka, Sveučilišna Avenija 4, 51000 Rijeka, Croatia; vtaksic@ffri.uniri.hr (V.T.); acosic@uniri.hr (A.Ć.P.)

**Keywords:** emotional literacy, emotional competence, emotion vocabulary, children

## Abstract

Emotional Literacy (EL) is a well-designed, field-tested curriculum that enhances social, emotional, and academic learning. A total of 565 students, 53% female, from 17 elementary schools, participated in the study. Approximately half of the students participated in the eight-week-long EL program, while the other half was in control conditions. Both the experimental and control group fulfilled the same set of trait and ability emotional intelligence measures in three timepoints: pre-, immediately after, and six months after the program. The effect of the EL program was different for girls and boys at different measurement points. Boys placed in experimental group improved their scores at both post-treatment measurements, meaning that they rated themselves and felt more emotionally competent after being a part of the EL program. Emotional understanding improved consistently with time, measured with both the Vocabulary of Emotions Test (VET) and Test of Emotional Understanding (TEU), regardless of the participation in the EL program. The effect of maturation was slightly more visible in girls, and girls had consistently better scores on the VET and TEU tests than boys. The feedback from school psychologists working with children was positive; they agreed that the children responded well to the activities and willingly participated.

## 1. Introduction

Under the umbrella term of emotional intelligence, introduced to the scientific community in the present form by Salovey and Mayer at the beginning of the 1990s [1,2], and popularized by Goleman in 1995 [3], there are other, similar constructs, including the emotional quotient [4,5], emotional competence [3,6] and emotional literacy [7,8]. Similar to emotional competence and in opposition to emotional intelligence, the term emotional literacy indicates that emotionally intelligent behavior can be taught. It has primarily been used in the area of education by emotional intelligence propagators associated with the Collaborative for Academic, Social and Emotional Learning (CASEL).

Emotional literacy refers to the ability to understand others’ emotions or the way others feel, and is a fundamental and important aspect of EI. Lately, this term has been replaced by the term social–emotional learning or SEL, with the word “learning” deliberately used to emphasize the fact that the acquisition of skills and attitudes is a process [9]. Elias et al. [10] defined SEL as the process of acquiring core competencies to recognize and manage emotions, set and achieve positive goals, appreciate the perspectives of others, establish and maintain positive relationships, make responsible decisions, and handle interpersonal situations constructively. These competencies are then utilized to improve adjustment and academic performance, and result in more positive social behaviors, fewer conduct problems, less emotional distress, and better grades and achievement test scores [11,12].

The Emotional Literacy (EL) program that was implemented in this study is a predecessor of the RULER program. The RULER was designed based on the Mayer and Salovey [13] four-branch model of emotional intelligence. It includes skill-building lessons and activities that can be used to recognize emotions in oneself and others, understand the causes and consequences of emotions, label emotions with an accurate and diverse vocabulary, and express and regulate emotions in socially appropriate ways, i.e., the RULER skills [14]. EL program teaches children from 5th–7th grade to label, understand and regulate emotions, thoughts, and actions in both themselves and others, using the “feeling words curriculum” [1]. The curriculum can be used as a stand-alone program or it can be introduced to students as part of their general literacy studies in any content area, i.e., vocabulary, comprehension, and oral and written expression, incorporating it as part of the normal lesson plan [1]. The feeling words for the program were carefully selected from lists of emotion terms and lexicons of behavioral terms that elicit a wide range of feeling in children. Included on the list are both “positive” and “negative” feeling terms, as well as behavioral terms that elicit an emotional reaction (such as motivation or commitment). Emotional Literacy engages the intellectual, emotional, and social aspects of individuals by using activities such as self-reflection, an analysis of academic material and current events, classroom discussions, interaction with family members, artistic designs, and creative writing assignments. Using different activities for interaction with feeling words leads to a thorough and complete understanding, as well as connecting them to personal and other people’s experience [1].

Research into the benefits of SEL programs found that social and emotional learning in school-aged children (both the ones implemented in schools and extracurricular programs) is improving social and emotional skills, attitudes about self and others, connection to school, positive social behavior and academic performance; they also reduced student’s conduct problems and emotional distress [15,16]. Programs are effective for children with and without identified emotional or behavioral problems. Academic performance was improved by 11 to 17 percentile points (as indicated by grades and standardized test scores) across the three large-scale reviews of research based on 317 studies involving more than 320,000 participants [16]. A 2017 meta-analysis investigated the long-term effects of SEL programs and showed that the academic performance of students exposed to SEL programs was, on average, 13 percentile points higher than their non-SEL peers [17]. The authors concluded that the effects of SEL programs are both positive and long-lasting.

Most of the theories that explain behavioral changes share a central concept of self-efficacy [18,19]. Bandura defined self-efficacy as an individual’s beliefs in one’s abilities to organize and execute the course of actions required to achieve a desired result. Self-efficacy beliefs are among the most important determinants of behavior, as the motivation to act is diminished if a person does not believe that he or she has the ability to perform and coordinate the actions necessary to produce results [20]. The three most important domains in the life of school-aged child and adolescents are emotional, social, and academic self-efficacy [20]. Petrides and Furnham [21], in their trait model of emotional intelligence, equated EI with the term emotional self-efficacy. Kirk, Schutte and Hine [22], on the other hand, asserted that emotional self-efficacy is one aspect of trait EI, but not equivalent to it. They defined it as a belief in one’s emotional functioning capabilities. Although these authors see emotional self-efficacy as overlapping with the emotional intelligence construct, the RULER approach sees them as distinct and builds on the social cognitive theory and the concept of self-efficacy to explain how the change in the behavior initiated by SEL occurs.

The effectiveness of the SEL program, like Emotional Literacy, has not been extensively studied in Croatia. Although several SEL programs were implemented in Croatian schools, there are only a few published studies [23,24]. The aim of our study was to examine the effects of a short-term Emotional Literacy program on students’ emotional intelligence (especially emotional vocabulary and emotional understanding), as well as self-reports of emotional competencies and social, emotional, and academic self-efficacy.

## 2. Method

### 2.1. Participants

In total, 565 students from 17 elementary schools situated in different regions of Croatia participated in all three measurement sessions. Children attended the 5th and 6th grades. Approximately half of the students participated in the eight-week-long Emotional Literacy program, while the other half was in the control condition, meaning that they were given the same instruments as the experimental group but had no extra activities (Table 1). Every school had one class in the experimental condition and one in the control condition.

### 2.2. Instruments

Both the experimental and control group filled out the same set of measures: The Emotional Skills and Competence Questionnaire—Children’s form (ESCQ-C) [25,26]; The Self-Efficacy Questionnaire for Children (SEQ-C) [27,28], Vocabulary of Emotions Test (VET) [29], and Test of Emotional Understanding (TEU) [30].

*The Emotional Skills and Competence Questionnaire—Children’s form* [25,26] is a measure of self-perceived emotional competences, measuring three components: self-perceived ability to perceive and understand emotions, to express and label emotions, and to manage and regulate emotions (of self and others). It has 21 items, divided into four subscales, and a 5-point answer scale (1—*never* to 5—*always*). In the original version, ESCQ [26,31] has 45 items, divided into three subscales, but in the children’s version, additional items were added in the manage and regulate emotion subscales to differentiate between regulation of one’s own emotions and the emotions of others [25]. The scale has good psychometric properties, and is reliable for subscales ranging from 0.76 to 0.82 [25]. The reliability coefficients obtained in our study for all used measures are shown in Table 2.

*The Self-Efficacy Questionnaire for Children* (SEQ-C) [27,28] measures *social self-efficacy* (perceived abilities in peer relationships and assertiveness); *academic self-efficacy* (perceived ability to manage one’s own learning behavior and master academic subjects); and *emotional self-efficacy* (the perceived ability to cope with negative emotions). It has 24 items, and each item is scored on a 5-point scale (1—*not at all* to 5—*very well*). The questionnaire was translated into Croatian by Vulić-Prtorić et al. [32].

*Vocabulary of Emotions Test* (VET) [29] measures the understanding of emotions, as a component of emotional intelligence from the Mayer and Salovey [13] hierarchical model. The test consists of 35 words. The first word is the target word, followed by six adjectives with similar meanings, and the respondent must choose one alternative, which is closest in meaning to the target word. A correct answer on the test is based on definitions given in a dictionary of Croatian language [33]. The test has been used in various pieces of research and has shown good psychometric properties, with a reliability coefficient of α = 0.91 [29].

*Test of Emotional Understanding* (TEU) [30] is an ability measure of emotional understanding, also defined within the Mayer–Salovey model of emotional intelligence [13]. It is an objective maximum-performance, multiple-choice test, with theoretically defined correct answers, based on Roseman’s [34] structural model of emotions. A test based on the same rationale has been developed by MacCann and Roberts [35]. The TEU consists of a description of 42 situations (three situations for each of the 14 different emotions). Each situation represents a specific combination of appraisal dimensions from Roseman’s structural model. The task of the subject is to recognize which emotion would arise from each of these situations. In previous research, it had a reliability coefficient of 0.76 [36]. A short version, with 16 items, was used in this study.

### 2.3. Procedure

After receiving a short education about the *Emotional Literacy* program, school psychologists were contacted with a request to participate in the validation study. Psychologist from 17 schools answered positively, and a booklet with detailed information regarding the validation procedures was sent to their e-mail addresses. Although authors [1] recommend that the EL program is incorporated into the regular school curriculum and implemented throughout the school year, due to organizational problems and limited possibilities to interfere with the school programs, we were able to implement only one part of the program. School psychologists were given detailed information about the procedures and activities they should apply with children. They selected two classes from their school: one for experimental and one for control conditions. The problems linked to group allocation are elaborated in the *Limitation and Future directions*.

The EL program validated in the study consisted of eight words/emotions (four pleasant—self-esteem, empathy, enthusiasm, and pride—and four unpleasant—isolation, stress, quilt, and anxiety). There are six steps for every word, which create a complete and holistic understanding of words. During sessions with children, school psychologists went through all eight feeling-words in the same order and same six steps, so that the program was implemented in the same way in all schools.

Upon receiving the necessary permissions, a set of questionnaires was given to all participants (1st measurement point). Then, half of the children participated in the EL program, and others had only regular school subjects. Immediately after the last session of the program, all children filled out questionnaires (regardless of their participation in the program) and completed them again after 6 months. Therefore, we had three measurement points, before the implementation of the EL program (at the beginning of the school year), immediately after the last session of the program, and at the end of school year (approximately 5 months after the sessions).

Permission for this study was obtained from Ethics Committee for Scientific Research from the Faculty of Humanities and Social Studies, University of Rijeka, Croatia.

## 3. Results

This study used a pre-test–post-test design, extended across nine months (from September to June), during the 2017/2018 academic year. Pre-test data were collected in September 2017 in a 1-h session before the beginning of the program. Post-test data were collected on two occasions, in January 2018 (immediately after the end of the program) and in June 2018 (approximately 5 months after the program). At all three timepoints, all participants (experimental and control groups) completed the surveys.

Table 3 reports the means and standard deviations for each outcome at baseline, separately for both groups and according to gender.

Table 4 reports the means and standard deviations for girls and boys in the experimental and control group at Times 2 and 3.

To check if participation in the Emotional Literacy program had an effect on the measured variables, we conducted three-way mixed ANOVA with one within-group (time) and two between-group factors (experimental/control group and gender). We summarized all the findings in Table 5.

First, we checked the homogeneity of variance for all used measures, with Levene’s test. We also checked the sphericity with Mauchly’s sphericity test and applied the appropriate Huynh–Feldt correction of degrees of freedom. The results are presented in Table 5.

**Figure 1:**
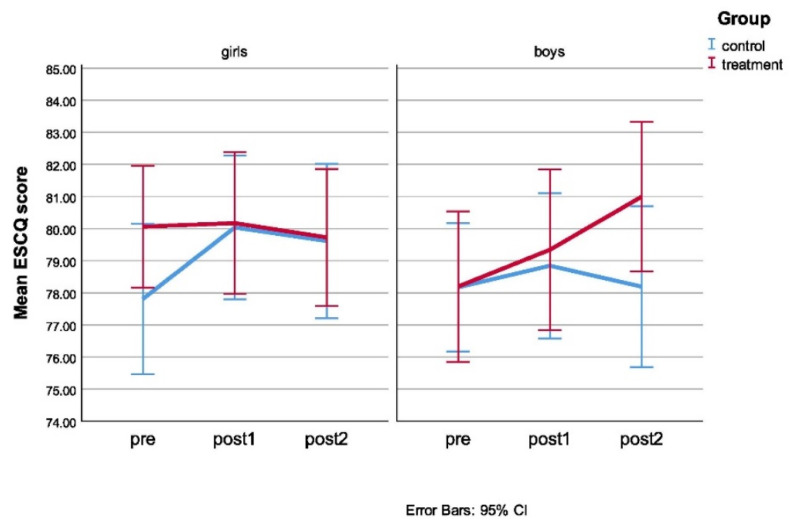
Mean ESCQ-C scores as a function of gender and group placement measured at different timepoints.

For the Emotional Skills and Competence Questionnaire (ESCQ-C) a statistically significant three-way interaction between group, gender and time was obtained, which means that simple group–gender interactions are different at different timepoints (Figure 1). As can be seen in Figure 1, girls in both the treatment and control group showed no difference in ESCQ-C scores on both post-test measurements. Boys, on the other hand, achieved more when they were placed in the treatment group than the ones that were in the control group. Bonferroni’s alpha level correction of 0.017 was used to assess the statistical significance of the simple two-way interaction. No statistically significant gender–group interactions were obtained at any of the test timepoints.

**Figure 2:**
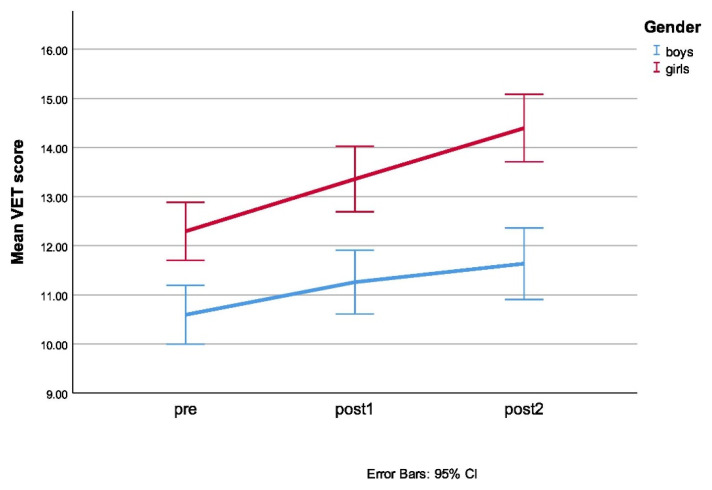
Mean VET scores for girls and boys at different measurement points.

Regarding the results for the Vocabulary of Emotions Test (VET), no statistically significant three-way interaction between group, gender, and time was obtained. A statistically significant two-way interaction between time and gender was obtained, as can be seen in Figure 2.

Other two-way interactions were not significant. The statistical significance of a simple main effect was accepted at a Bonferroni-adjusted alpha level of 0.017. There was a statistically significant simple main effect of gender at all three testing timepoints, meaning that girls had better results on the Vocabulary of Emotions Test on all three measurement times, regardless of their placement in the experimental/control group.

When results of the Test of Emotional Understanding (TEU) and the Self-efficacy questionnaire (SEQ-C) were compared, no statistically significant three-way interaction of group, gender, and time was obtained. There were no significant two-way interactions.

To conclude, the effects of maturation (change over time regardless of the participation in the Emotional Literacy program) were obtained for Vocabulary of Emotions Test (VET) and Test of Emotional Understanding (TEU). Emotional understanding as measured with both tests improved consistently with time. There were no changes over time seen on the Emotional Skills and Competence Questionnaire (ESCQ-C) and the Self-Efficacy Questionnaire (SEQ-C).

Emotional competence measured with ESCQ-C showed different group x gender patterns at different timepoints. Although two-way interactions were not statistically significant, boys placed in the treatment group showed an improvement in post-test measurements and this effect was not seen for girls. There were no effects of gender or treatment on self-efficacy. A significant interaction between gender and VET was found at different timepoints, suggesting that the effect of maturation was slightly more visible in girls. Additionally, girls had consistently better scores on the VET test than boys. Test of Emotional Understanding also showed that girls had better emotional understanding.

Since this program is expected to develop emotional literacy, answers on the Vocabulary of Emotions Test were analyzed in more detail. Although participation in the program did not have an effect on the student’s emotional vocabulary, our results suggest that it is very important to keep educating students on feeling-words. As can be seen in Table 2 and Table 3, students, on average, know the meaning of from 11 to 13 words (out of 35, with girls scoring slightly better than boys). In the first measurement point, less than 20% of answers were correct for eight words: *touching*, *spiritless* (only 9% of students knew the correct answer), *monotonous*, *lonely*, *intolerant*, *pessimistic*, *tolerant*, and *shocked*. The easiest word was *abandoned*, with 61% of answers being correct. After the program (Time 2 measurement), correct answers were under 30% for only four words, and over 50% of answers were correct for ten words. Similar results were obtained for Time 3 (5 months after the program).

## 4. Discussion

The aim of our study was to explore the effects and possible benefits of the Emotional Literacy program on students’ emotional and social competences, emotional understanding and vocabulary, and perceived emotional, social, and academic self-efficacy. We found a significant three-way interaction (time *x* group *x* gender) in the Emotional Skills and Competence Questionnaire, which showed that the effect of the Emotional Literacy program was different for girls and boys at different measurement points. Boys placed in the experimental group improved their scores at both post-treatment measurements, meaning that they rated themselves and felt more emotionally competent after being a part of the EL program—an effect that was not found for girls. Although the 2-way group of *x* gender interactions at all measurement points were not significant, it seems that there is an indication that the program may be more efficient for boys.

Our results also indicate that girls outperform boys on different aspects of emotional competence. Girls obtained better results on the Vocabulary of Emotions Test (VET) and Test of Emotional Understanding (TEU). Surprisingly, we found no gender differences in terms of perceived emotional competence and self-efficacy, although different studies show that girls usually perceive themselves better than boys [37,38]. Fernández-Berrocal, Cabello, Castillo and Extremera [39] believe that gender differences are mediated by age; therefore, we should be cautious when concluding that gender is a determining variable in EI, unless we have thoroughly analyzed the potential interaction with other variables. Dylman et al. [40] found that girls produced more emotion words than boys in the vocabulary emotion task, and this may be connected to the finding that girls generally express their emotions more than boys (e.g., [41]).

Besides gender differences, maturation effects were also found for both tests measuring ability (emotional understanding and emotional vocabulary), suggesting that students had better emotional understanding (TEU), and emotion vocabulary (VET) at the end of the school year, compared to their results at the beginning of the school year. It is important to emphasize that the observed changes were small, and one school year is not a sufficient period for significant variations in EI to occur among adolescents. Esnaola et al. [42] analyzed the development of the different dimensions of emotional intelligence in adolescents over one school year and in a cross-sectional study involving 484 adolescents of both genders from the six school years, between year 1 of Spanish secondary school (age 12–13) and year 2 of the Spanish Baccalaureate (age 17–18). Their findings indicate that, except for the stress management dimension in the female sample group, none of the dimensions of emotional intelligence undergo substantial changes in relation to age. Regarding EI development, some authors suggest that age has no significant effect on EI (e.g., [43]), while others imply a direct relationship between age and EI levels in the sense that EI is learnt through life experience [44,45,46]. Few studies have focused on EI development in adolescence. Keefer, Holden, and Parker [47] reported non-variance in three (intrapersonal, interpersonal and adaptability) of the four scales of the Emotional Quotient Inventory: Young Version Short [44] between the ages of 12 and 18. Regarding changes in accordance with age, the authors state that the findings present a complex panorama, with varying decreasing, increasing and steady patterns depending on age and the different specific scales.

Although we found no other significant three-way interaction, we believe that the necessity of the program is visible in some other respects. For example, students, on average, have a poor emotional vocabulary and know the meaning of from 11 to 13 words (out of 35). More than 80% of the answers to some words were wrong, which is very indicative and shows that greater attention should be paid to teaching students emotion-related or feeling words. The ability to correctly understand and use emotion words is an essential part of communication and vital part of emotional and social competence, both of which are particularly important in the period of early adolescence, when forming social relationships becomes of special value. Ekman [48] states that, without being able to name feelings, it becomes harder to distinguish them, think about them, or make plans regarding them. The ability to use a correct and rich emotional vocabulary is the basis of emotional competence and emotional intelligence, and a first step for the successful perception, recognition, and regulation of emotions [49]. Not knowing emotion (or feelings) words can lead one to interpret the behavior of others as intentionally hurtful and result in behaviors that can cause social isolation [50]. Discriminating among affective states such as anger, sadness, frustration, or happiness requires a vocabulary of emotion words. In a classroom that incorporates the learning of emotion words into its curriculum, and where teachers help students to acquire a rich and varied emotion vocabulary, fewer problematic behaviors and more enjoyable peer social relations can be expected [15,16].

## 5. Limitations and Future Directions

In our study we found no significant effects of the Emotional Literacy program on either perceived or objectively measured emotional competence. There are several possible explanations for those findings and the most probable is that the program was too short for any significant change to occur. Due to the tough schedule in Croatian schools, it was possible to arrange only eight sessions, on average (covering eight emotions), which were held after regular school classes. The authors of the program strongly suggest that the program should be implemented as a part of regular school activities, and that it should last for at least the whole school year.

Another major limitation is that participants were not assigned randomly to a condition; a school psychologist decided which class would participate in the program, and which class would only participate in the research part and fill in the questionnaires. Several reasons could have influenced this decision—the school psychologist could have chosen the class which they thought would benefit the most from the program (with students with some emotional or behavioral problems) or a class they knew it would be easy to work with. They also may have had to choose a class which was not engaged in other school programs and had room for some extra activities.

Moreover, several people were engaged in facilitating the sessions, and although they all had received the same detailed plan on how to work with students, we must assume that there were differences in their approach. An experimental design in which a classes are chosen based on similar characteristics would improve the power of the findings. Several other factors should also be controlled, such as the size of a school, or urban–rural location of a school. Although there were eight schools situated in cities and nine schools from smaller towns or rural areas, from different parts of the country, we did not control for the regional development or socioeconomic status of students and their parents, which could have had an effect on the results.

Nevertheless, the feedback received from the school psychologists working with children was positive: they agreed that the children responded well to the activities and willingly participated. Additionally, especially important information for on-going curriculum reform in Croatia was obtained: the social and emotional learning courses must be planned for at least two academic years and should be part of the various regular subjects (fine art, literature, history, etc.).

## Figures and Tables

**Table 1:** Description of the study participants.

Participants	N (%)	M	SD
Female	297 (52.5%)	11.25	0.69
Male	268 (47.5%)	11.25	0.72
Experimental group	293 (51.8%)	11.26	0.68
Control group	272 (48.2%)	11.23	0.73
Total	565	11.25	0.71

**Table 2:** Cronbach Alpha reliability coefficients for used measures in three measurement timepoints.

Measure	Time 1	Time 2	Time 3
The Emotional Skills and Competence Questionnaire—Children’s form (ESCQ-C)	0.82	0.88	0.90
The Self-Efficacy Questionnaire for Children (SEQ-C)	0.82	0.82	0.82
Vocabulary of Emotions Test (VET)	0.77	0.80	0.83
Test of Emotional Understanding (TEU)	0.70	0.75	0.75

**Table 3:** Pre-test means and standard deviations for girls and boys in the experimental and control group at baseline (first measurement point).

TIME 1	Control Group (*N* = 272)		Experimental Group (*N* = 293)	
	Girls (*n* = 143)	Boys (*n* = 129)	Girls (*n* = 154)	Boys (*n* = 139)
	*M* (*SD*)	*M* (*SD*)	*M* (*SD*)	*M* (*SD*)
Vocabulary of Emotions Test	12.55 (5.22)	10.75 (4.81)	12.05 (5.18)	10.44 (5.15)
Test of Emotional Understanding	11.97 (2.73)	10.97 (3.08)	11.66 (2.57)	10.27 (3.48)
Self-efficacy	90.05 (11.64)	90.62 (11.97)	90.65 (12.39)	92.10 (12.37)
Emotional Skills and Competence Questionnaire	77.81 (14.19)	78.17 (11.47)	80.06 (11.94)	78.19 (13.89)

**Table 4:** Post intervention group differences on all outcomes.

TIME 2	Control Group (*N* = 272)		Experimental Group (*N* = 293)	
	Girls (*n* = 143)	Boys (*n* = 129)	Girls (*n* = 154)	Boys (*n* = 139)
	*M* (*SD*)	*M* (*SD*)	*M* (*SD*)	*M* (*SD*)
Vocabulary of Emotions Test	13.38 (5.80)	11.37 (5.48)	13.33 (5.85)	11.14 (5.28)
Test of Emotional Understanding	11.78 (2.79)	11.01 (3.34)	11.74 (2.74)	10.22 (3.48)
Self-efficacy	89.23 (11.71)	90.93 (12.87)	90.93 (11.02)	90.99 (12.39)
Emotional Skills and Competence Questionnaire	80.03 (13.53)	78.84 (12.91)	80.16 (13.81)	79.34 (14.89)
**TIME 3**	**Control group** **(*N* = 272)**		**Experimental group** **(*N* = 293)**	
	**Girls** **(*n* = 143)**	**Boys** **(*n* = 129)**	**Girls** **(*n* = 154)**	**Boys** **(*n* = 139)**
	***M*** **(*SD*)**	***M*** **(*SD*)**	***M*** **(*SD*)**	***M*** **(*SD*)**
Vocabulary of Emotions Test	14.34 (6.39)	11.55 (6.04)	14.43 (5.67)	11.71 (6.08)
Test of Emotional Understanding	12.28 (2.53)	11.07 (3.40)	12.14 (2.39)	11.25 (3.56)
Self-efficacy	88.72 (13.31)	90.39 (12.66)	89.81 (12.11)	89.83 (13.68)
Emotional Skills and Competence Questionnaire	79.61 (14.54)	78.19 (14.37)	79.72 (13.40)	80.99 (13.91)

**Table 5:** Main ANOVA findings.

Main ANOVA Findings				
	Homogeneity of variance (Leven’s test)	Mauchly’s sphericity test	Three-way interaction (group × gender × time)	Two-way interactions and main effects
Emotional Skills and Competence Questionnaire (ESCQ-C)	*p* < 0.05 at 1st time-point; *p* > 0.05 at all 2nd and 3rd time points	χ^2^(2) = 9.322, *p* = 0.009	*F*(1.985, 1103.556) = 3.787, *p* = 0.023, partial η^2^ = 0.007, ε = 0.992	Two-way interaction (gender × group): 1st time-point *F*(1, 559) = 1.039, *p* = 0.309 2nd time-point *F*(1, 558) = 0.024, *p* = 0.877 3rd time-point *F*(1, 561) = 1.287, *p* = 0.257
Vocabulary of Emotions Test (VET)	*p* > 0.05 at all three time points	χ^2^(2) = 14.252, *p* = 0.001	*F*(1.968, 1104.140) = 0.150, *p* = 0.858, partial η^2^ = 0.000, ε = 0.984	Two-way interaction (time × gender): *F*(1.968, 1104.140) = 4.605, *p* = 0.011; Simple main effect of gender at all three time points of testing: *F*(1, 561) = 15,646, *p* = 0.000; *F*(1, 561) = 19.539, *p* = 0.000; *F*(1, 561) = 29.328, *p* = 0.000 The effects of maturation: *F*(2, 1695) = 11.134, *p* < 0.05, partial η^2^ = 0.013 Gender differences: *F*(1, 561) = 28.42, *p* < 0.01, partial η^2^ = 0.048
Test of Emotional Understanding (TEU)	*p* > 0.05 at all three time points	χ^2^(2) = 9.400, *p* = 0.009	*F*(1.985, 1113.403) = 2.510, *p* = 0.082, partial η^2^ = 0.004, ε = 0.992)	No significant two-way interactions; The effects of maturation: *F*(2, 1695) = 4.733, *p* < 0.05, partial η^2^ = 0.006 Gender effects: *F*(1, 561) = 28.42, *p* < 0.01, partial η^2^ = 0.048
Self-efficacy questionnaire (SEQ-C)	*p* > 0.05 at all three time points	χ^2^(2) = 5.770, *p* = 0.056	*F*(2, 676) = 0.284, *p* = 0.753	No significant two-way interactions (*p* > 0.05)

## Data Availability

The data presented in this study are available on reasonable request from the corresponding author.
